# The chronification of post-COVID condition associated with neurocognitive symptoms, functional impairment and increased healthcare utilization

**DOI:** 10.1038/s41598-022-18673-z

**Published:** 2022-08-25

**Authors:** Mayssam Nehme, Olivia Braillard, François Chappuis, CoviCare Study Team, Mayssam Nehme, Olivia Braillard, Pauline Vetter, Delphine S. Courvoisier, Frederic Assal, Frederic Lador, Lamyae Benzakour, Matteo Coen, Ivan Guerreiro, Gilles Allali, Christophe Graf, Jean-Luc Reny, Silvia Stringhini, Hervé Spechbach, Frederique Jacquerioz, Julien Salamun, Guido Bondolfi, Dina Zekry, Paola M. Soccal, Riccardo Favale, Stéphane Genevay, Kim Lauper, Philippe Meyer, Nana Kwabena Poku, Agathe Py, Basile N. Landis, Thomas Agoritsas, Marwène Grira, José Sandoval, Julien Ehrsam, Simon Regard, Camille Genecand, Aglaé Tardin, Laurent Kaiser, François Chappuis, Idris Guessous, Idris Guessous

**Affiliations:** 1 Division of Primary Care Medicine, Geneva University Hospitals, Geneva, Switzerland; 2 Faculty of Medicine, University of Geneva, Geneva, Switzerland; 3 Division of Tropical and Humanitarian Medicine, Geneva University Hospitals, Geneva, Switzerland; 4 Division of Infectious Diseases, Geneva University Hospitals, Geneva, Switzerland; 5 Geneva Center for Emerging Viral Diseases, Geneva University Hospitals, Geneva, Switzerland; 6 Division of Laboratory Medicine, Laboratory of Virology, Geneva University Hospitals, Geneva, Switzerland; 7 Quality of Care Division, Medical Directorate, Geneva University Hospitals, Geneva, Switzerland; 8 Cantonal Health Service, General Directorate for Health, Geneva, Switzerland; 9 Division of Neurology, Geneva University Hospitals, Geneva, Switzerland; 10 Division of Pulmonary Medicine, Geneva University Hospitals, Geneva, Switzerland; 11 Division of Psychiatry, Geneva University Hospitals, Geneva, Switzerland; 12 Division of General Internal Medicine, Geneva University Hospitals, Geneva, Switzerland; 13 Leenaards Memory Center, Lausanne University Hospital and University of Lausanne, Lausanne, Switzerland; 14 Department of Rehabilitation and Geriatrics, Geneva University Hospitals, Geneva, Switzerland; 15 Division of Rheumatology, Geneva University Hospitals, Geneva, Switzerland; 16 Division of Cardiology, Geneva University Hospitals, Geneva, Switzerland; 17 Division of Otolaryngology, Geneva University Hospitals, Geneva, Switzerland; 18 Department of Oncology, Geneva University Hospitals, Geneva, Switzerland; 19 Department of Medical Information Sciences, Geneva University Hospitals, Geneva, Switzerland; 20 Division of Emergency Medicine, Geneva University Hospitals, Geneva, Switzerland

**Keywords:** SARS-CoV-2, Prognosis, Public health, Quality of life

## Abstract

Post-COVID condition is prevalent in 10–35% of cases in outpatient settings, however a stratification of the duration and severity of symptoms is still lacking, adding to the complexity and heterogeneity of the definition of post-COVID condition and its oucomes. In addition, the potential impacts of a longer duration of disease are not yet clear, along with which risk factors are associated with a chronification of symptoms beyond the initial 12 weeks. In this study, follow-up was conducted at 7 and 15 months after testing at the outpatient SARS-CoV-2 testing center of the Geneva University Hospitals. The chronification of symptoms was defined as the continuous presence of symptoms at each evaluation timepoint (7 and 15 months). Adjusted estimates of healthcare utilization, treatment, functional impairment and quality of life were calculated. Logistic regression models were used to evaluate the associations between the chronification of symptoms and predictors. Overall 1383 participants were included, with a mean age of 44.3 years, standard deviation (SD) 13.4 years, 61.4% were women and 54.5% did not have any comorbidities. Out of SARS-CoV-2 positive participants (n = 767), 37.0% still had symptoms 7 months after their test of which 47.9% had a resolution of symptoms at the second follow-up (15 months after the infection), and 52.1% had persistent symptoms and were considered to have a chronification of their post-COVID condition. Individuals with a chronification of symptoms had an increased utilization of healthcare resources, more recourse to treatment, more functional impairment, and a poorer quality of life. Having several symptoms at testing and difficulty concentrating at 7 months were associated with a chronification of symptoms. COVID-19 patients develop post-COVID condition to varying degrees and duration. Individuals with a chronification of symptoms experience a long-term impact on their health status, functional capacity and quality of life, requiring a special attention, more involved care and early on identification considering the associated predictors.

## Introduction

Post-COVID condition is increasingly recognized with symptoms that may persist several weeks^1^ to months^2^. The World Health Organization consensus definition describes post-COVID condition as symptoms persisting at least 3 months after the infection, after excluding other causes^3^. The prevalence of post-COVID condition varies between 10 and 35% of infected individuals^4^ and can reach up to 70% in patients post-hospitalization^5^. In a recent systematic review of 57 studies, post-covid condition manifested mostly through fatigue, pulmonary, neurologic and mental health long-term consequences^6^. Data are now emerging on the direct impact of COVID-19 on the brain among other organ systems. A recent study showed changes on the brain imaging of infected patients on average 141 days after their infection, mostly in the limbic system and correlated with a larger cognitive decline compared to SARS-CoV-2 negative individuals^7^. In a different study, brain structural changes were associated with functional modifications affecting different brain networks depending on whether the patients were aware of their deficits or not^8^. Another study suggested astrocytic impairment potentially underlying reported neurological post-COVID symptoms^9^, and brain tissue autopsies of SARS-CoV-2 infected non-human primates showed multiple evidence of neurologic damage including in animals that did not develop severe respiratory disease, potentially providing insight into the neurological manifestations of post-COVID condition^10^.

Without current treatment options, and as more evidence is gathered to explain the underlying pathophysiology, time and the natural evolution of symptoms accompanied by interdisciplinary care, rehabilitation and the management of daily activities remain the cornerstone of therapy^11–13^. While the prevalence of persistent symptoms decreases with time^5,14–16^, a subset of patients develop chronic symptoms that may impact them on the long-term. Thus, infected individuals could potentially be categorized into three groups: acute infection without post-COVID condition, acute infection with post-COVID condition, and acute infection with post-COVID condition and a chronification of symptoms. The latter group may experience increased functional impairment, with a debilitating tail of the pandemic that can last for years. This has been described after the 1918 influenza pandemic^17^, the severe acute respiratory syndrome (SARS) outbreak in 2003^18^, and the Middle East Respiratory Syndrome (MERS) in 2012^19^, among other viruses. In post-COVID condition, studies have already suggested an increased functional impairment in healthcare workers and in the general population related to the direct effects of the virus^20,21^. With chronic disability, patients may be sicker for a long time with a poorer quality of life, and require more healthcare resources and treatment^22^.

The subset of patients with a chronification of symptoms along with potential consequences have not been studied yet in post-COVID condition, focusing initially on the overall prevalence without a stratification of severity, duration and long-term impact. Additionally, some evidence suggests that female sex, the number of symptoms in the acute phase, age and body-mass index (BMI) are associated with post-COVID condition^23,24^. However, it is not yet clear which risk factors could be associated with a chronification of symptoms.

This study aims to describe and evaluate the evolution of symptoms at 7 and 15 months after SARS-CoV-2 infection, in order to determine the proportion of individuals with a chronification of symptoms and their impact on functional capacity, quality of life, and healthcare utilization as well as any potential predictors, compared to the general population, to infected individuals with post-COVID condition without a chronification of symptoms and to infected individuals without post-COVID condition.

## Methods

### Participants and study setting

Individuals tested for SARS-CoV-2 by Reverse Transcriptase Polymerase Chain Reaction (RT-PCR) at the outpatient center of the Geneva University Hospitals between October and December 2020 and who had an email address on file were contacted in July 2021 and January 2022 for follow-up. Inclusion criteria included a laboratory-confirmed test date at the Geneva University Hospitals, and being symptomatic at time of testing. Exclusion criteria included being asymptomatic at time of testing, having a positive test in between the laboratory confirmed test result at the Geneva University Hospitals and follow-up,  or having a reinfection less than 7 months prior to follow-up.

### Ethical approval and consent to participate

All individuals gave consent and the study was approved by the Cantonal Research Ethics Commission of Geneva, Switzerland (protocol number 2021-00389). All methods were performed in accordance with relevant guidelines and regulations.

### Data collection

Participants completed follow-up in July 2021 and January 2022. Follow-up included questions about self-rated health, symptoms at time of testing, evolution of symptoms since testing, persistent symptoms, symptoms intensity and frequency when present, functional capacity, productivity, quality of life, chronic treatment, and utilization of healthcare resources including hospitalizations, visits to the emergency room, visits to the primary care physician or other specialists. The follow-up survey instrument is presented in Supplement 1. Fatigue was assessed using the Chalder fatigue scale^25^ and the Eastern Cooperative Oncology Group (ECOG) performance scale^26^. Dyspnea was assessed using the modified Medical Research Council (mMRC) scale^27^ and the Nijmegen questionnaire^28^. Insomnia was assessed using the insomnia severity index (ISI)^29^, anxiety and depression were assessed using the hospital anxiety and depression scale (HAD)^30^. All remaining symptoms were assessed using a Likert scale with self-reported options of mild, moderate or severe. Quality of life was assessed using the 12-item short form survey (SF-12) questionnaire^31^. Self-rated health was assessed using the first question of the 12-item short form survey “How would you rate your general state of health prior to testing” with answers (1) excellent, (2) very good, (3) good, (4) poor, (5) very poor. Answers were then combined into “good to excellent”, and “poor to very poor”. Functional capacity was assessed using the Sheehan disability scale^32^. The Sheehan disability scale is a five-item questionnaire. The first three items are each graded from 0 (no impairment), 1–3 (mild impairment), 4–6 (moderate impairment), 7–9 (marked impairment), to 10 (extreme impairment) evaluating functional impairment in three domains: professional, social and family life. Each domain can be assessed separately, and a global impairment rating is derived by adding the three scores. The remaining two items of the five-item questionnaire evaluate the number of days lost and days with reduced productivity due to functional impairment in the week preceding the questionnaire.

The chronification of symptoms was defined as the continuous presence of symptoms at each evaluation timepoint (7 and 15 months). Groups of participants were defined as: (1) infected individuals with post-COVID condition and a chronification of symptoms (individuals reporting symptoms since the infection with symptoms present at each follow-up), (2) infected individuals with post-COVID condition without a chronification of symptoms (individuals reporting having symptoms for more than 12 weeks after the infection with symptoms present at the first follow-up and no symptoms at the second follow-up), (3) infected individuals without post-COVID condition (individuals reporting symptoms lasting less than 12 weeks after the infection), and (4) SARS-CoV-2 negative individuals. The inclusion of SARS-CoV-2 negative individuals aimed to compare the differential impact of post-COVID condition with a chronification of symptoms to individuals who were not infected, by introducing a group of individuals who lived through similar pandemic conditions but did not get infected. The goal was to determine the direct effect of the infection, and potentially evaluate an increasing impact proportional to the effect of the infection *versus* no infection.

### Data analysis

Statistical analysis was conducted using STATA v16.0. Descriptive analyses included percentages with comparisons using chi-square tests. A p-value of less than 0.05 was used for significance. Symptoms defining the presence of symptoms were any new symptom onset after SARS-CoV-2 infection including: fatigue, insomnia, headache, dyspnea, chest pain, palpitations, dizziness, difficulty concentrating, paresthesia, loss or change in smell, loss or change in taste, generalized pain, myalgia, arthralgia, fever, cough, digestive symptoms (nausea, vomiting, diarrhea, constipation, abdominal pain), and hair loss.

In order to determine the impact of the chronification of symptoms on individuals, estimates of healthcare utilization, treatment, functional impairment and quality of life were calculated and compared between the four defined groups. These estimates were adjusted for age, sex, physical activity, smoking status, vaccination status, hospitalization, self-rated health prior to testing, symptoms at testing and the following comorbidities: obesity or overweight, hypertension, diabetes, respiratory disease, cardiovascular disease, headache disorders, cognitive disorders, sleep disorders, depression, anxiety, hypothyroidism, rheumatologic disease, anemia, chronic pain or fibromyalgia, chronic fatigue syndrome, and irritable bowel syndrome.

In order to determine potential predictors of the chronification of symptoms, the group of individuals with post-COVID condition without a chronification of symptoms was compared to the group of individuals with post-COVID condition with a chronification of symptoms. Logistic regression models were used to evaluate the associations between the chronification of symptoms and the following predictors: age, sex, having several symptoms at time of testing, and symptoms at 7 months (fatigue, difficulty concentrating, headache, dizziness, insomnia, loss or change in smell, loss or change in taste, myalgia or arthralgia, or dyspnea). Adjusted odds ratios (aOR) were adjusted for age, sex, profession, civil status (single, married, widowed/separated or divorced), number of symptoms at time of testing, vaccination status, hospitalization, and pre-existing comorbidities (cognitive disorders, headaches, depression, anxiety), based on previous studies evaluating risk factors for the chronification of symptoms in chronic fatigue syndrome^33–35^.

## Results

### Overall participants

**Figure 1:**
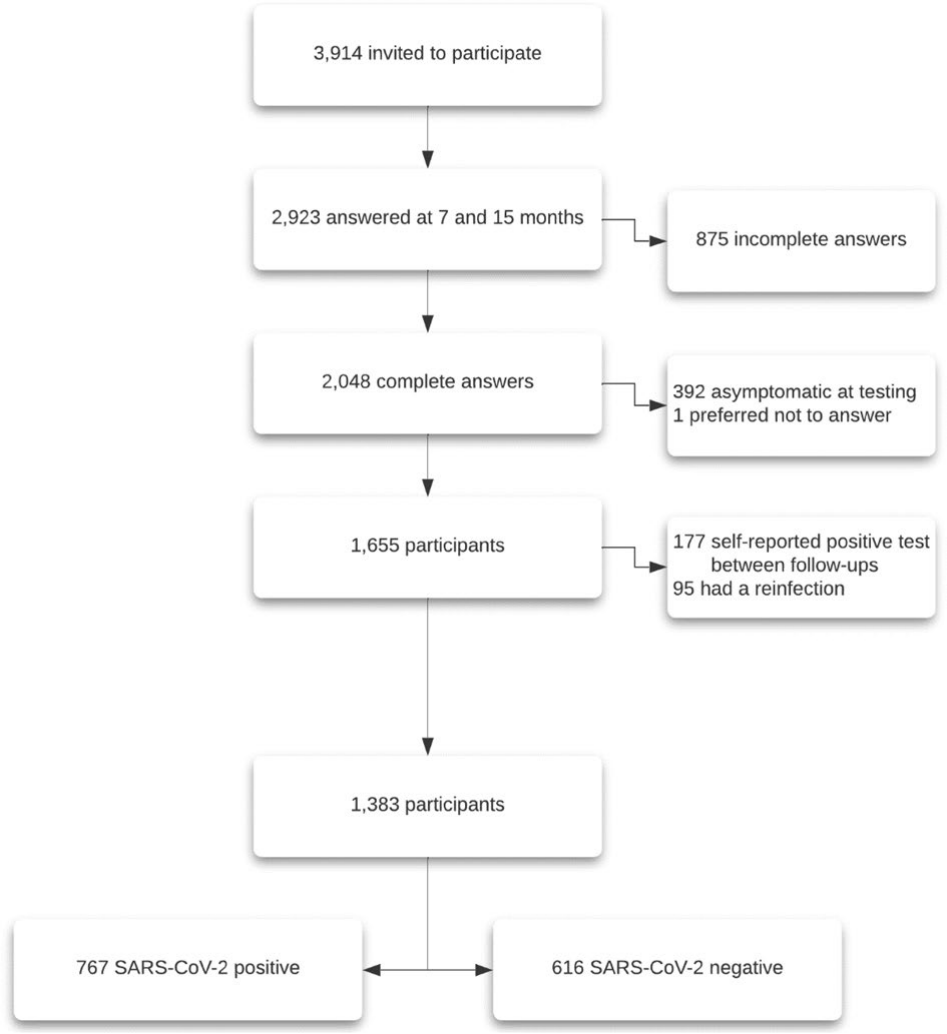
Flowchart with 1383 participants included out of 2923 who had a follow-up at 7 and 15 months. Out of the 1383 participants, 767 were SARS-CoV-2 positive and 616 were SARS-CoV-2 negative.

Out of 3914 participants in July 2021, 2923 had consented to both follow-ups (response rate 74.7%) and 2048 completed both follow-ups fully. Out of the 2048 participants (44.8% with a positive test result, 55.2% with a negative test result), 392 had no symptoms at time of testing, and 1 preferred not to answer, both groups were excluded from this study. Out of the remaining participants, 177 had a positive test result between their documented RT-PCR and the second follow-up and 95 had a reinfection between their initial positive test result and the second follow-up; both groups were excluded from this study. Overall 1383 participants were included, 767 had a positive test result and 616 had a negative test result (Fig. 1). The mean age of participants was 44.3 years, standard deviation (SD) 13.4 years; 61.4% were women and 54.5% did not have any comorbidities. In comparison, individuals who did not participate were 55.3% women, mean age was 41.3 years (SD 13.8); 46.2% had a SARS-CoV-2 positive test, 9.4% were hospitalized and 48.9% did not have any comorbidities. Further characteristics are shown in Table 1, stratification by group of participants is presented in Supplement 2.

**Table 1:** Baseline characteristics of participants (n = 1383).

	N (%)
**Age categories**	
Below 40 years	554 (40.1)
40–59 years	653 (47.2)
60 years and above	176 (12.7)
**Sex**	
Male	534 (38.6)
Female	849 (61.4)
**Education**	
Primary	39 (2.8)
Apprenticeship	158 (11.4)
Secondary	174 (12.6)
Tertiary	932 (67.4)
Other	61 (4.4)
Prefer not to answer	19 (1.4)
**RT-PCR test result**	
Negative	616 (44.5)
Positive	767 (55.5)
**Civil status**	
Single	265 (20.7)
In couple, not married	334 (26.1)
Married or registered partnership	528 (41.2)
Divorced or separated	130 (10.1)
Widowed	15 (1.2)
Other	10 (0.8)
**Living situation**	
Alone	265 (20.7)
Single parent with children	94 (7.3)
In couple, without children	325 (25.4)
In couple, with children	474 (37)
Cohabitation	123 (9.6)
**Work status**	
Salaried	909 (71.0)
Retired	105 (8.2)
Student or in training	93 (7.3)
Independent worker	64 (.05)
Homemaker	33 (2.6)
Unemployed	40 (3.1)
Disability	12 (0.9)
Other	24 (1.9)
**Work situation**	
Fixed term contract	114 (11.1)
Open-ended long term contract	817 (79.7)
Subsidized	3 (0.3)
Training	26 (2.5)
Other	65 (6.3)
**Profession**	
Unskilled workers	48 (3.5)
Skilled workers	238 (17.2)
Highly skilled workers	323 (23.4)
Professional-managers	438 (31.7)
Other	206 (14.9)
Prefer not to answer	20 (1.5)
**Smoking status**	
Never smoked	741 (53.6)
Current smoker	207 (15.0)
Ex-smoker, stopped independently of COVID-19	400 (28.9)
Ex-smoker, stopped because of COVID-19 infection	9 (0.7)
Prefer not to answer	26 (1.9)
**Physical activity**	
No physical activity	195 (14.1)
Partial physical activity	711 (51.4)
Complete physical activity	469 (33.9)
Prefer not to answer	8 (0.6)
**Body-mass index**	
Less than 18.5 kg/m^2^	38 (3.1)
Between 18.5–24.9 kg/m^2^	724 (58.7)
Between 25–29.9 kg/m^2^	351 (28.4)
Between 30–34.9 kg/m^2^	93 (7.5)
Between 35 and 40 kg/m^2^	28 (2.3)
**Symptoms at testing**	
Pauci-symptomatic	461 (33.3)
Several symptoms	922 (66.7)
**Vaccination status**	
No vaccination	183 (13.2)
1 dose	215 (15.5)
2 doses	455 (32.9)
3 doses	524 (37.9)
Prefer not to answer	6 (0.4)
Hospitalization	90 (6.5)
**Comorbidities**	
None	755 (54.6)
Obesity or overweight	275 (19.9)
Hypertension	144 (10.4)
Diabetes	26 (1.9)
Respiratory disease	74 (5.4)
Cardiovascular disease	40 (2.9)
Headache disorders^a^	230 (16.6)
Cognitive disorders^b^	117 (8.5)
Sleep disorders	249 (18.0)
Depression	116 (8.4)
Anxiety	149 (10.8)
Hyperthyroidism	12 (0.9)
Hypothyroidism	49 (3.5)
Anemia	63 (4.6)
Thromboembolic disease	16 (1.2)
Dysmenorrhea	18 (1.3)
Fibromyalgia or chronic pain	35 (2.5)
Chronic fatigue syndrome	99 (7.2)
Rheumatologic disorders^c^	186 (13.4)
Irritable bowel syndrome	98 (7.1)

^a^Headache disorders include migraine, tension headaches, and other types of headaches.

^b^Cognitive disorders include memory and attention deficit.

^c^Rheumatologic disorders include tendinitis, polymyalgia rheumatica, arthritis, and ankylosing spondylitis.

### Symptoms at 7 and 15 months

Out of SARS-CoV-2 positive participants (n = 767), 63.0% (n = 483) had no symptoms 7 months after their test (first follow-up median time 208 days, interquartile range IQR 194–221), and 37.0% (n = 284) still had symptoms 7 months after their test and were considered to have post-COVID condition. Out of individuals with post-COVID condition, 47.9% (n = 136) had a resolution of symptoms at the second follow-up (15 months after the infection), and 52.1% (n = 148) had persistent symptoms and were considered to have a chronification of their post-COVID condition. The most common remaining symptoms at 15 months were fatigue, difficulty concentrating, headache, insomnia, loss or change in smell, loss or change in taste, myalgia, arthralgia, and dyspnea.

### Utilitzation of resources and treatment

**Figure 2:**
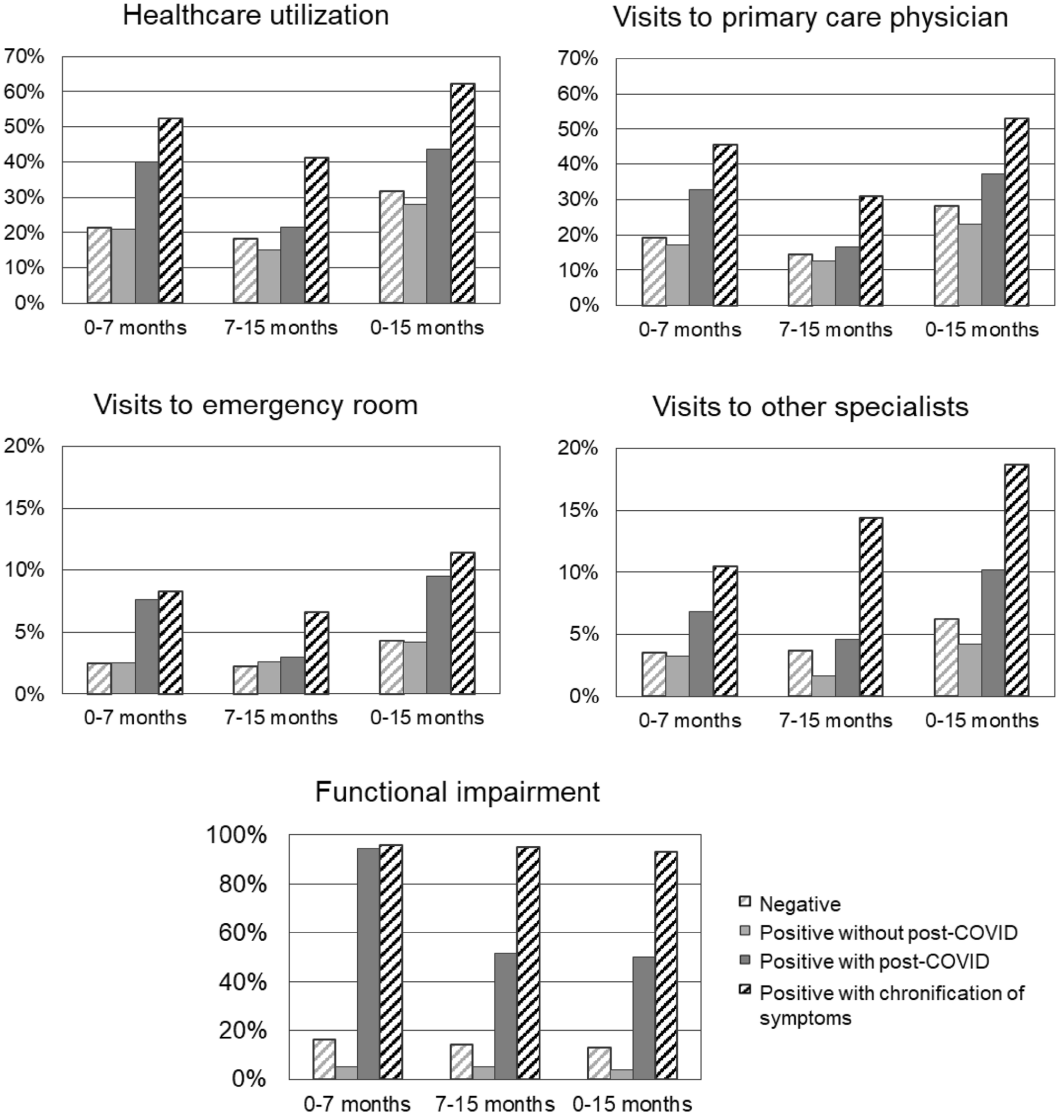
Adjusted frequency estimates of utilization of healthcare resources since test date, stratified by SARS-CoV-2 infection and duration of symptoms, including infected individuals without post-COVID condition, with post-COVID condition without a chronification of symptoms and with a chronification of symptoms (n = 1383). Healthcare utilization was defined as the presence of any of the visits to the primary care physician, the emergency room or other specialists since the test date. Chronification of symptoms was defined as the continuous persistence of symptoms, present at 7 and 15 months of follow-up. Symptoms defining the persistence of symptoms were any new symptom onset after SARS-CoV-2 infection including: fatigue, insomnia, headache, dyspnea, chest pain, palpitations, dizziness, difficulty concentrating, paresthesia, loss or change in smell, loss or change in taste, generalized pain, myalgia, arthralgia, fever, cough, digestive symptoms (nausea, vomiting, diarrhea, constipation, abdominal pain), and hair loss. Estimates were adjusted for age, sex, physical activity, smoking status, vaccination status, hospitalization, self-rated health prior to testing, symptoms at testing and the following comorbidities: obesity or overweight, hypertension, diabetes, respiratory disease, cardiovascular disease, headache disorders, cognitive disorders, sleep disorders, depression, anxiety, hypothyroidism, rheumatologic disease, anemia, chronic pain or fibromyalgia, chronic fatigue syndrome, and irritable bowel syndrome.

Individuals with a chronification of symptoms had an increased utilization of healthcare resources since their test date (62.2% at 15 months), when compared to infected individuals with post-COVID without chronification of symptoms (43.6%), infected individuals without post-COVID (27.9%), and SARS-CoV-2 negative individuals (31.6%), with details presented in Fig. 2. The increased utilization of resources included visits to the emergency room, visits to the primary care physician and visits to specialists. Individuals with a chronification of symptoms had more recourse to treatment overall (10.0% reported no treatment at all since their test date) compared to infected individuals with post-COVID without a chronification of symptoms (13.3% without any treatment), infected individuals without post-COVID (21.4% without any treatment), and SARS-CoV-2 negative individuals (12.6% without any treatment). SARS-CoV-2 negative individuals had a higher usage of anti-depressants and anxiolytics when compared to the other groups. More details on treatment consumption are presented in Table 2.

**Table 2:** Adjusted frequency estimates of treatment, functional impairment and quality of life in SARS-CoV-2 positive individuals with and without chronification of symptoms, and SARS-CoV-2 negative individuals (n = 1383).

	Negative (n = 616)	Positive without post-COVID symptoms (n = 483)	Positive with post-COVID symptoms without chronification of symptoms (n = 136)	Positive with chronification of symptoms (n = 148)	P-value
	% (95%)	% (95%)	% (95%)	% (95%)	
**Treatment since testing**					
None	12.6 (11.9–13.4)	21.4 (20.2–22.5)	13.3 (11.7–14.9)	10.0 (8.8–11.2)	< 0.001
Paracetamol	67.7 (66.7–68.8)	60.8 (59.5–62.1)	73.4 (71.3–75.4)	72.3 (70.2–74.5)	< 0.001
Anti-inflammatories	46.6 (45.5–47.7)	35.5 (34.3–36.7)	41.4 (39.2–43.5)	39.4 (37.3–41.4)	< 0.001
Aspirin	8.2 (7.8–8.7)	7.8 (7.3–8.4)	8.1 (7.2–9.0)	8.2 (7.1–9.4)	0.007
Inhaled sprays	9.6 (8.4–10.8)	5.3 (4.4–6.2)	8.6 (6.4–10.9)	10.5 (8.0–13.0)	< 0.001
Systemic steroids	3.0 (2.7–3.3)	3.6 (3.1–4.0)	3.8 (3.0–4.6)	6.1 (5.1–7.0)	< 0.001
Anticoagulation	1.8 (1.5–2.1)	2.7 (2.2–3.2)	2.0 (1.5–2.6)	2.0 (1.5–2.5)	0.011
Antidepressants	11.2 (9.7–12.6)	4.3 (3.2–5.3)	6.4 (3.9–8.8)	8.3 (5.9–10.7)	< 0.001
Sleeping medication	5.2 (4.4–6.1)	1.9 (1.3–2.4)	9.7 (6.9–12.5)	6.9 (5.1–8.7)	< 0.001
Anxiolytics	8.3 (7.2–9.3)	4.1 (3.3–4.8)	6.9 (4.9–8.9)	4.5 (3.3–5.7)	< 0.001
Vitamin D	27.4 (26.5–28.4)	21.2 (20.4–21.9)	34.3 (32.0–36.5)	38.0 (35.9–40.0)	< 0.001
Vitamin C	19.4 (18.8–20.0)	16.4 (15.9–16.9)	30.3 (28.9–31.7)	26.6 (25.4–27.9)	< 0.001
Vitamin B12	11.6 (11.2–12.1)	9.5 (9.2–9.9)	18.3 (17.1–19.5)	14.3 (13.3–15.2)	< 0.001
Zinc	12.4 (11.9–13.0)	12.1 (11.6–12.6)	17.6 (16.3–18.9)	18.1 (16.7–19.4)	< 0.001
Other treatment	5.9 (5.6–6.3)	5.1 (4.7–5.4)	5.2 (4.6–5.8)	6.8 (6.1–7.6)	< 0.001
Physical therapy	23.7 (22.6–24.8)	16.2 (15.4–17.1)	17.3 (15.3–19.2)	24.0 (22.3–25.8)	< 0.001
**Impact at 15 months**					
Functional impairment^a^	12.5 (11.8–13.2)	5.0 (4.6–5.3)	43.7 (41.2–46.2)	95.6 (95.2–96.0)	< 0.001
1 or more days lost or with reduced productivity in the past week	23.6 (22.7–24.5)	23.8 (22.9–24.7)	16.8 (15.5–18.2)	46.9 (44.7–49.2)	< 0.001
**Quality of life**^**b**^					
Physical health score	51.0 (50.7–51.2)	52.7 (52.5–52.9)	51.9 (51.5–52.3)	46.1 (45.7–46.5)	< 0.001
Mental health score	40.7 (40.5–40.8)	41.9 (41.7–42.0)	39.7 (39.3–40.0)	40.2 (39.9–40.5)	< 0.001

Estimates were adjusted for age, sex, physical activity, smoking status, vaccination status, hospitalization, self-rated health prior to testing, symptoms at time of testing and the following comorbidities: obesity or overweight, hypertension, diabetes, respiratory disease, cardiovascular disease, headache disorders, cognitive disorders, sleep disorders, depression, anxiety, hypothyroidism, rheumatologic disease, anemia, chronic pain or fibromyalgia, chronic fatigue syndrome, and irritable bowel syndrome.

^a^Functional impairment was calculated using the Sheehan disability scale^32^.

^b^Quality of life was assessed using the SF-12 scale^31^.

### Functional impairment and quality of life

Individuals with a chronification of symptoms had consistently more functional impairment at each follow-up (93.0% at 15 months), compared to individuals with post-COVID condition without a chronification of symptoms who had an improvement in functional capacity between the first and second follow-up and individuals without post-COVID condition who did not report functional impairment (Fig. 2). They also had an increased frequency of days lost or with reduced productivity at work (46.9% compared to 16.8% in post-COVID individuals without a chronification of symptoms, 23.8% in SARS-CoV-2 positive individuals without post-COVID condition and 23.6% in SARS-CoV-2 negative individuals). Details are presented in Table 2. In addition, SARS-CoV-2 positive individuals with a chronification of symptoms had a poorer quality of life evidenced by lower physical health scores on the SF-12 scale (mean 46.1 in group 1, compared to 51.9 in post-COVID individuals without a chronification of symptoms, 52.7 in SARS-CoV-2 positive individuals without post-COVID condition, 51.0 in SARS-CoV-2 negative individuals). The mental health scores on the SF-12 scale were low across all four groups.

### Predictors of chronification

**Figure 3:**
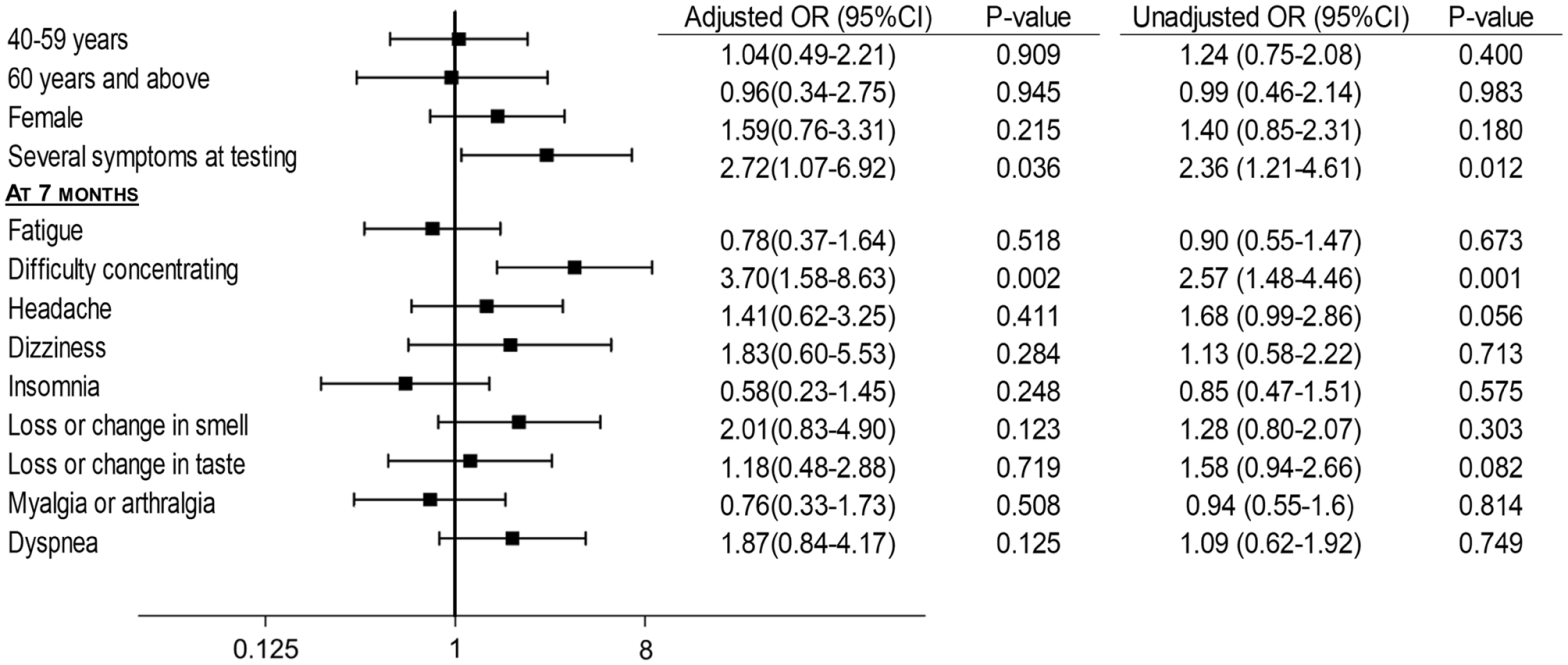
Associations between predictors and chronification of symptoms in SARS-CoV-2 positive individuals with post-COVID condition (n = 284). Individuals who had symptoms at 7 months and no symptoms at 15 months were considered to have post-COVID condition without chronification. Individuals with symptoms at 7 months and 15 months were considered to have post-COVID condition with chronification. Symptoms defining the presence of symptoms were any new symptom onset after SARS-CoV-2 infection including: fatigue, insomnia, headache, dyspnea, chest pain, palpitations, dizziness, difficulty concentrating, paresthesia, loss or change in smell, loss or change in taste, generalized pain, myalgia, arthralgia, fever, cough, digestive symptoms (nausea, vomiting, diarrhea, constipation, abdominal pain), and hair loss. Odds ratios were adjusted for age, sex, profession, civil status (single, married, widowed/separated or divorced), symptoms at time of testing, number of symptoms at 7 months, nature of symptom at 7 months (fatigue, difficulty concentrating, headache, dizziness, loss or change in smell, loss or change in taste, insomnia, myalgia, arthralgia, dyspnea), vaccination status, hospitalization, and pre-existing comorbidities (cognitive disorders, headaches, depression, anxiety.

When comparing individuals with post-COVID condition without chronification to individuals with post-COVID condition with chronification, results showed an independent association between having difficulty concentrating at 7 months and the chronification of symptoms (aOR 3.70; 1.58–8.63). The chronification of symptoms was also associated with having several symptoms at time of testing (aOR 2.72; 1.07–6.92), and was not associated with age, sex, or any other symptom at 7 months (Fig. 3).

## Discussion

Post-COVID symptoms improve in almost 50% of individuals with post-COVID condition between 7 and 15 months after testing. Yet, the chronification of symptoms defined as the continuous presence of symptoms at each follow-up and beyond 12 months after the infection is prevalent in 19.3% (148/767) of infected individuals. The chronification of symptoms is predicted by neurologic symptoms and leads to an increased utilization of healthcare resources, as well as functional impairment.

A stratification of post-COVID condition, taking into account the nature and duration of post-COVID symptoms is important in addressing this condition, as all patients may not require the same treatment or management. The chronification of symptoms largely increases the utilization of healthcare resources, including visits to the emergency room and outpatient visits, as well as the need for more chronic treatment. The increased utilization of healthcare resources and treatment options remains significant after adjusting for sociodemographic variables and comorbidities. Of note, SARS-CoV-2 negative individuals had a higher use of anti-depressants and anxiolytics when compared to the other groups. One hypothesis is that people with more anxiety or depression might have opted to stay home or be less socially active during the pandemic thus decreasing their risk of SARS-CoV-2 infection^36,37^. The increased use of healthcare resources and treatment potentially leads to compounded individual and public health effects due to the chronification of symptoms.

The impact of the chronification of symptoms on individuals and on society is manifested by an increased functional impairment, more days lost or with reduced productivity at work. These results show that a proportion of individuals will suffer from debilitating effects, long after their infection. Individuals with a chronification of symptoms also suffer from a poorer quality of life. While mental health scores were overall low in all study groups compared to the average of 50 in the general population^31^, the physical health scores were markedly lower in the group of individuals with a chronification of symptoms, indicating potential physical limitations in their everyday life. While awaiting for potential treatment options, patients with potential risk factors of chronification should be identified and managed early in order to potentially reduce the impact on daily life as much as possible.

Risk factors for the chronification of symptoms may include having several symptoms at time of testing and the presence of cognitive symptoms evidenced by a difficulty concentrating. A recent study described the longitudinal evolution of symptoms over 12 months and suggested neurologic symptoms such as paresthesia might increase with time^16^. This is in line with our hypothesis that the chronification of symptoms may be driven by a neurological process. In comparison, risk factors for other chronic conditions similar to post-COVID have been reported such as age, sex, education levels, depression or anxiety found to be associated with an increased risk of chronic fatigue syndrome^33–35,38^. However, these studies have shown contradictory results showing an association with lower income^34^, but also with middle-high income leading to more diagnosis^38^. Studies also showed an association of chronic fatigue syndrome with younger^33^, and older age^35^. These factors were not associated with a chronification of symptoms in our study. Also comparatively, patients with post-COVID condition after hospitalization might exhibit more respiratory symptoms^6^, which was not as evident in our study. To date, the definition of post-COVID condition includes both outpatient and inpatient settings however the manifestation of disease might be different and so could the predicting factors.

The underlying mechanisms of post-COVID are not yet understood. Four hypotheses remain at the forefront of explanations of post-COVID condition including viral persistence, dysbiosis^39^, an autoimmune response^40^ or a dysregulated inflammatory response^39,41^. Studies have shown that inflammatory markers are elevated in post-COVID individuals^42^, autoantibodies during the acute phase could be correlated with long-term symptoms, and smaller studies have detected persistent viral particles on gastrointestinal biopsies^43,44^. In addition to learning about the mechanisms of post-COVID condition, it is paramount to understand the mechanisms leading to a chronification of symptoms. Comparatively, the chronification of symptoms in chronic fatigue syndrome shows that immune dysregulation or a low-level chronic inflammation might be potential contributors^45^.

Limitations include ascertainment bias in online follow-ups based on a survey format, as well as including only laboratory-confirmed results. This was a decision based on the importance to objectively differentiate SARS-CoV-2 from other viruses or diseases as much as possible, however could potentially miss taking into account false negative results^46^ or individuals who did not have timely access to testing. In addition, the chronification of symptoms is a newly defined concept that was elaborated specifically in this study context, to be validated by further studies. Similarly, reports of cognitive signs and disorders did not include a validated questionnaire which would have required an in-person assessment. Including a validated questionnaire would better define cognitive symptoms in addition to the ones used for fatigue, dyspnea and sleep disorders. Finally, more women participated in the study raising the issue of the generalizability. Age and sex were adjusted for in the regression models and frequency estimates.

In conclusion, this study shows that COVID-19 patients develop post-COVID condition to varying degrees and durations, and a subset of individuals might have a chronification of their symptoms, potentially predicted by neurologic manifestations, and with a long-term impact including poor quality of life, increased functional impairment and an increased utilization of healthcare resources. The risks of the chronification of symptoms and functional impairment should be assessed early on, taking into account identified predictors and providing specific and more involved care for these individuals to mitigate long-term consequences.

## Supplementary Information

Supplementary Information 1.

Supplementary Information 2.

Supplementary Information 3.

## Data Availability

The datasets used and analyzed during the current study are available from the corresponding author upon reasonable request.
