# Supplementary material for: The chronification of post-COVID condition associated with neurocognitive symptoms, functional impairment and increased healthcare utilization
Source: Sci Rep. 2022 Aug 25;12:14505. doi: 10.1038/s41598-022-18673-z (PMC9403954; doi:10.1038/s41598-022-18673-z)
Supplement: Supplementary file 2 — Supplementary Information 2. [file 41598_2022_18673_MOESM2_ESM.docx]

Supplement 2. Baseline characteristics stratified by SARS-CoV-2 infection and duration of symptoms, including infected individuals without post-COVID condition, with post-COVID condition without a chronification of symptoms and with post-COVID condition with a chronification of symptoms (n=1,383)

|  | **Negative**  **(n=616)** | **Positive without post-COVID condition**  **(n=483)** | **Positive with post-COVID condition**  **(n=136)** | **Positive with post-COVID condition with a chronification of symptoms**  **(n=148)** | **Total**  **(n=1,383)** | **P-value** |
| --- | --- | --- | --- | --- | --- | --- |
|  | N(%) | N(%) | N(%) | N(%) | N(%) |  |
| **Age categories** |  |  |  |  |  | 0.799 |
| Below 40 years | 255(41.4) | 188(38.9) | 58(42.6) | 53(35.8) | 554(40.1) |  |
| 40-59 years | 284(46.1) | 231(47.8) | 60(44.1) | 78(52.7) | 653(47.2) |  |
| 60 years and above | 77(12.5) | 64(13.3) | 18(13.2) | 17(11.5) | 176(12.7) |  |
| **Sex** |  |  |  |  |  | <0.001 |
| Male | 188(30.5) | 244(50.5) | 55(40.4) | 47(31.8) | 534(38.6) |  |
| Female | 428(69.5) | 239(49.5) | 81(59.6) | 101(68.2) | 849(61.4) |  |
| **Education** |  |  |  |  |  | 0.615 |
| Primary | 16(2.6) | 15(3.1) | 3(2.2) | 5(3.4) | 39(2.8) |  |
| Apprenticeship | 60(9.7) | 62(12.8) | 21(15.4) | 15(10.1) | 158(11.4) |  |
| Secondary | 78(12.7) | 55(11.4) | 20(14.7) | 21(14.2) | 174(12.6) |  |
| Tertiary | 428(69.5) | 326(67.5) | 85(62.5) | 93(62.8) | 932(67.4) |  |
| Other | 24(3.9) | 20(4.1) | 6(4.4) | 11(7.4) | 61(4.4) |  |
| Prefer not to answer | 10(1.6) | 5(1.0) | 1(0.7) | 3(2.0) | 19(1.4) |  |
| **Civil status** |  |  |  |  |  | 0.708 |
| Single | 125(21.8) | 86(19.3) | 28(24.6) | 26(17.6) | 265(20.7) |  |
| In couple, not married | 159(27.7) | 106(23.8) | 29(25.4) | 40(27.0) | 334(26.1) |  |
| Married or registered partnership | 214(37.3) | 201(45.1) | 48(42.1) | 65(43.9) | 528(41.2) |  |
| Divorced or separated | 64(11.1) | 45(10.1) | 8(7.0) | 13(8.8) | 130(10.1) |  |
| Widowed | 7(1.2) | 5(1.1) | 1(0.9) | 2(1.4) | 15(1.2) |  |
| Other | 5(0.9) | 3(0.7) | 0(0) | 2(1.4) | 10(0.8) |  |
| **Living situation** |  |  |  |  |  | 0.063 |
| Alone | 132(23) | 79(17.8) | 28(24.6) | 26(17.6) | 265(20.7) |  |
| Single parent with children | 49(8.5) | 21(4.7) | 7(6.1) | 17(11.5) | 94(7.3) |  |
| In couple, without children | 140(24.4) | 114(25.6) | 28(24.6) | 43(29.1) | 325(25.4) |  |
| In couple, with children | 204(35.5) | 179(40.2) | 42(36.8) | 49(33.1) | 474(37) |  |
| Cohabitation | 49(8.5) | 52(11.7) | 9(7.9) | 13(8.8) | 123(9.6) |  |
| **Work status** |  |  |  |  |  | 0.498 |
| Salaried | 395(69.1) | 320(71.7) | 81(71.1) | 113(76.4) | 909(71) |  |
| Retired | 48(8.4) | 36(8.1) | 11(9.6) | 10(6.8) | 105(8.2) |  |
| Student or in training | 34(5.9) | 40(9) | 9(7.9) | 10(6.8) | 93(7.3) |  |
| Independent worker | 35(6.1) | 20(4.5) | 4(3.5) | 5(3.4) | 64(5.0) |  |
| Homemaker | 17(3.0) | 12(2.7) | 2(1.8) | 2(1.4) | 33(2.6) |  |
| Unemployed | 24(4.2) | 10(2.2) | 2(1.8) | 4(2.7) | 40(3.1) |  |
| Disability | 5(0.9) | 2(0.4) | 2(1.8) | 3(2.0) | 12(0.9) |  |
| Other | 14(2.4) | 6(1.3) | 3(2.6) | 1(0.7) | 24(1.9) |  |
| **Work situation** |  |  |  |  |  | 0.126 |
| Fixed term contract | 57(12.9) | 33(9) | 10(10.8) | 14(11.3) | 114(11.1) |  |
| Long term contract | 350(79.2) | 294(80.3) | 71(76.3) | 102(82.3) | 817(79.7) |  |
| Subsidized | 3(0.7) | 0(0) | 0(0) | 0(0) | 3(0.3) |  |
| Training | 7(1.6) | 14(3.8) | 2(2.2) | 3(2.4) | 26(2.5) |  |
| Other | 25(5.7) | 25(6.8) | 10(10.8) | 5(4.0) | 65(6.3) |  |
| **Profession** |  |  |  |  |  | 0.373 |
| Unskilled workers | 19(3.1) | 21(4.3) | 4(2.9) | 4(2.7) | 48(3.5) |  |
| Skilled workers | 112(18.2) | 76(15.7) | 25(18.4) | 25(16.9) | 238(17.2) |  |
| Highly skilled workers | 141(22.9) | 104(21.5) | 33(24.3) | 45(30.4) | 323(23.4) |  |
| Professional-Managers | 202(32.8) | 163(33.7) | 36(26.5) | 37(25.0) | 438(31.7) |  |
| Other | 93(15.1) | 63(13.0) | 25(18.4) | 25(16.9) | 206(14.9) |  |
| Prefer not to answer | 9(1.5) | 7(1.4) | 3(2.2) | 1(0.7) | 20(1.5) |  |
| **Smoking status** |  |  |  |  |  |  |
| Never smoked | 302(49) | 280(58.0) | 76(55.9) | 83(56.1) | 741(53.6) |  |
| Current smoker | 118(19.2) | 58(12.0) | 16(11.8) | 15(10.1) | 207(15.0) | <0.001 |
| Ex-smoker, stopped independently of COVID-19 | 185(30.0) | 139(28.8) | 33(24.3) | 43(29.1) | 400(28.9) |  |
| Ex-smoker, stopped because of COVID-19 infection | 2(0.3) | 2(0.4) | 3(2.2) | 2(1.4) | 9(0.7) |  |
| Prefer not to answer | 9(1.5) | 4(0.8) | 8(5.9) | 5(3.4) | 26(1.9) |  |
| **Physical activity** |  |  |  |  |  | <0.001 |
| No physical activity | 103(16.7) | 57(11.8) | 14(10.3) | 21(14.2) | 195(14.1) |  |
| Partial physical activity | 322(52.3) | 214(44.3) | 80(58.8) | 95(64.2) | 711(51.4) |  |
| Full physical activity | 188(30.5) | 210(43.5) | 40(29.4) | 31(20.9) | 469(33.9) |  |
| Prefer not to answer | 3(0.5) | 2(0.4) | 2(1.5) | 1(0.7) | 8(0.6) |  |
| **Body-mass index** |  |  |  |  |  | 0.703 |
| Less than 18.5 kg/m2 | 13(2.4) | 15(3.5) | 3(2.7) | 7(4.9) | 38(3.1) |  |
| Between 18.5-24.9 kg/m2 | 333(60.4) | 253(59) | 62(55.9) | 76(53.1) | 724(58.7) |  |
| Between 25-29.9 kg/m2 | 152(27.6) | 123(28.7) | 34(30.6) | 42(29.4) | 351(28.4) |  |
| Between 30-34.9 kg/m2 | 39(7.1) | 32(7.5) | 10(9.0) | 12(8.4) | 93(7.5) |  |
| Between 35 and 40 kg/m2 | 14(2.5) | 6(1.4) | 2(1.8) | 6(4.2) | 28(2.3) |  |
| **Self-rated health prior to testing** |  |  |  |  |  | 0.008 |
| Poor | 60(9.7) | 22(4.6) | 7(5.2) | 11(7.4) | 100(7.2) |  |
| Good | 556(90.3) | 461(95.4) | 128(94.8) | 137(92.6) | 1,282(92.8) |  |
| **Symptoms at testing** |  |  |  |  |  | <0.001 |
| Pauci-symptomatic | 286(46.4) | 126(26.1) | 31(22.8) | 18(12.2) | 461(33.3) |  |
| Several symptoms | 330(53.6) | 357(73.9) | 105(77.2) | 130(87.8) | 922(66.7) |  |
| **Vaccination status** |  |  |  |  |  | <0.001 |
| No vaccination | 65(10.6) | 71(14.7) | 35(25.7) | 12(8.1) | 183(13.2) |  |
| 1 dose | 59(9.6) | 84(17.4) | 36(26.5) | 36(24.3) | 215(15.5) |  |
| 2 doses | 174(28.2) | 183(37.9) | 39(28.7) | 59(39.9) | 455(32.9) |  |
| 3 doses | 316(51.3) | 144(29.8) | 24(17.6) | 40(27) | 524(37.9) |  |
| Prefer not to answer | 2(0.3) | 1(0.2) | 2(1.5) | 1(0.7) | 6(0.4) |  |
| **Hospitalization** | 40(6.5) | 32(6.6) | 6(4.4) | 12(8.1) | 90(6.5) | 0.845 |
| **Comorbidities** |  |  |  |  |  |  |
| None | 322(52.3) | 293(60.7) | 68(50) | 72(48.6) | 755(54.6) | 0.008 |
| Obesity or overweight | 131(21.3) | 79(16.4) | 31(22.8) | 34(23.0) | 275(19.9) | 0.106 |
| Hypertension | 63(10.2) | 48(9.9) | 16(11.8) | 17(11.5) | 144(10.4) | 0.899 |
| Diabetes | 9(1.5) | 11(2.3) | 1(0.7) | 5(3.4) | 26(1.9) | 0.288 |
| Respiratory disease | 42(6.8) | 16(3.3) | 8(5.9) | 8(5.4) | 74(5.4) | 0.084 |
| Cardiovascular disease | 13(2.1) | 16(3.3) | 5(3.7) | 6(4.1) | 40(2.9) | 0.448 |
| Headache disorders* | 111(18) | 66(13.7) | 25(18.4) | 28(18.9) | 230(16.6) | 0.189 |
| Cognitive disorders** | 51(8.3) | 29(6.0) | 18(13.2) | 19(12.8) | 117(8.5) | 0.010 |
| Sleep disorders | 127(20.6) | 61(12.6) | 27(19.9) | 34(23) | 249(18.0) | 0.002 |
| Depression | 66(10.7) | 31(6.4) | 11(8.1) | 8(5.4) | 116(8.4) | 0.037 |
| Anxiety | 80(13.0) | 39(8.1) | 18(13.2) | 12(8.1) | 149(10.8) | 0.033 |
| Hyperthyroidism | 6(1.0) | 5(1.0) | 0(0) | 1(0.7) | 12(0.9) | 0.684 |
| Hypothyroidism | 23(3.7) | 14(2.9) | 3(2.2) | 9(6.1) | 49(3.5) | 0.245 |
| Anemia | 34(5.5) | 20(4.1) | 6(4.4) | 3(2.0) | 63(4.6) | 0.297 |
| Thromboembolic disease | 7(1.1) | 5(1.0) | 2(1.5) | 2(1.4) | 16(1.2) | 0.972 |
| Dysmenorrhea | 8(1.3) | 7(1.4) | 1(0.7) | 2(1.3) | 18(1.3) | 0.935 |
| Fibromyalgia or chronic pain | 25(4.1) | 3(0.6) | 2(1.5) | 5(3.4) | 35(2.5) | 0.003 |
| Chronic fatigue syndrome | 50(8.1) | 24(5.0) | 8(5.9) | 17(11.5) | 99(7.2) | 0.031 |
| Rheumatologic disorders*** | 91(14.8) | 49(10.1) | 21(15.4) | 25(16.9) | 186(13.4) | 0.059 |
| Irritable bowel syndrome | 49(8.0) | 30(6.2) | 9(6.6) | 10(6.8) | 98(7.1) | 0.720 |
| *Headache disorders include migraine, tension headaches, and other types of headaches;  **Cognitive disorders include memory and attention deficits;  ***Rheumatologic disorders include tendinitis, polymyalgia rheumatica, arthritis, and ankylosing spondylitis | | | | | | |
|  | | | | | | |
