# Supplementary material for: The chronification of post-COVID condition associated with neurocognitive symptoms, functional impairment and increased healthcare utilization
Source: Sci Rep. 2022 Aug 25;12:14505. doi: 10.1038/s41598-022-18673-z (PMC9403954; doi:10.1038/s41598-022-18673-z)
Supplement: Supplementary file 3 — Supplementary Information 3. [file 41598_2022_18673_MOESM3_ESM.docx]

**Main authors**

Mayssam Nehme^1*^, Olivia Braillard^1^, François Chappuis^2,3^, CoviCare Study team**, and Idris Guessous^1,2^

**Consortium list separate from main authors**

**CoviCare Study Team : Pauline Vetter^4,5,6^, Delphine S. Courvoisier^7,8^, Frederic Assal^2,9^, Frederic Lador^10^, Lamyae Benzakour^11^, Matteo Coen^12^, Ivan Guerreiro^10^, Gilles Allali^9,13^, Christophe Graf^14^, Jean-Luc Reny^12^, Silvia Stringhini^1,2^, Hervé Spechbach^1^, Frederique Jacquerioz^1,3,5^, Julien Salamun^1^, Guido Bondolfi^11^, Dina Zekry^14^, Paola M. Soccal^10^, Riccardo Favale^11^, Stéphane Genevay^15^, Kim Lauper^15^, Philippe Meyer^16^, Nana Kwabena Poku^16^, Agathe Py^16^, Basile N. Landis^17^, Thomas Agoritsas^12^, Marwène Grira^1^, José Sandoval^18^, Julien Ehrsam^1,19^, Simon Regard^8,20^, Camille Genecand^8^, Aglaé Tardin^8^, Laurent Kaiser^4,5,6^

1. Division of Primary Care Medicine, Geneva University Hospitals, Geneva, Switzerland
2. Faculty of Medicine, University of Geneva, Geneva, Switzerland
3. Division of Tropical and Humanitarian Medicine, Geneva University Hospitals, Geneva, Switzerland
4. Division of Infectious diseases, Geneva University Hospitals, Geneva, Switzerland
5. Geneva Center for Emerging Viral Diseases, Geneva University Hospitals, Geneva, Switzerland
6. Division of Laboratory Medicine, Laboratory of Virology, Geneva University Hospitals, Geneva, Switzerland
7. Quality of Care Division, Medical Directorate, Geneva University Hospitals, Geneva, Switzerland
8. Cantonal Health Service, General Directorate for Health, Geneva, Switzerland
9. Division of Neurology, Geneva University Hospitals, Geneva, Switzerland
10. Division of Pulmonary Medicine, Geneva University Hospitals, Geneva, Switzerland
11. Division of Psychiatry, Geneva University Hospitals, Geneva, Switzerland
12. Division of General Internal Medicine, Geneva University Hospitals, Geneva, Switzerland
13. Leenaards Memory Center, Lausanne University Hospital and University of Lausanne, Lausanne, Switzerland
14. Department of rehabilitation and geriatrics, Geneva University Hospitals, Geneva, Switzerland
15. Division of Rheumatology, Geneva University Hospitals, Geneva, Switzerland
16. Division of Cardiology, Geneva University Hospitals, Geneva, Switzerland
17. Division of Otolaryngology, Geneva University Hospitals, Geneva, Switzerland
18. Department of Oncology, Geneva University Hospitals, Geneva, Switzerland
19. Department of Medical Information Sciences, Geneva University Hospitals, Geneva, Switzerland
20. Division of Emergency Medicine, Geneva University Hospitals, Geneva, Switzerland
